# Porto-systemic shunt using adrenal vein as a conduit; an alternative procedure for spleno – renal shunt

**DOI:** 10.1186/1471-2482-7-7

**Published:** 2007-06-07

**Authors:** Unal Aydin, Pinar Yazici, Murat Kilic

**Affiliations:** 1 Ege University School of Medicine, Department of Surgery, Izmir, Turkey

## Abstract

**Background:**

Currently, portal hypertension is still big problem for the patients with serious liver diseases. Variceal bleeding is one of the most important complications of portal hypertension. In case of failure of endoscopic and combined medical treatments, surgical decompressive shunts are required. We emphasized an alternative splenorenal shunt procedure using adrenal vein as a conduit.

**Case presentation:**

A 26-year-old male suffered from recurrent variceal bleeding was considered for surgical therapy. Although we planned to perform a distal splenorenal shunt procedure, it was observed to be difficult. Therefore left adrenal vein was used as a conduit between left renal vein and splenic vein after splenic artery was ligated. He did well and was discharged from the hospital on the postoperative day 6. In the follow up period for nine months, endoscopic and ultrasonographic examinations were normal.

**Conclusion:**

We concluded that, in case of failure to perform distal splenorenal shunt due to technical problems, alternative porto-systemic shunt procedure using the adrenal vein as a vascular conduit can be safely employed.

## Background

In spite of new therapeutic modalities such as pharmacologic, endoscopic (sclerotherapy or band ligation), and transjugular intrahepatic portosystemic shunt (TIPSS), surgical decompressive shunts still have an important place in the treatment of portal hypertension (PHT). One of the serious complications of PHT is variceal bleeding which can lead to death. Nevertheless, the success rate of the endoscopic and combined medical treatments is about 50%. A few surgical procedures such as Sugiura procedure for esophageal varices and Hassab-Paquet (A complete gastroesophageal devascularization with splenectomy) procedure for gastric varices have also been performed for PHT [1,2]. In case of failure of those procedures, surgical methods should be considered. Distal spleno-renal shunt (DSRS) and/or portocaval shunt are still regarded as the most popular surgical procedures in these patients [3]. Type of the surgical shunt should be chosen in the light of prognosis, anatomic variations and surgical experience.

The technique of DSRS was first described by Warren in 1967. DSRS is originally end to side anastomosis between splenic and left renal veins [4]. End to side anastomosis may be unfavorable due to scar tissues or inappropriate diameter between the two veins. A few cases had been used adrenal vein for DSRS in the English literature in Pubmed search [5-9]. In this case report, we emphasized a successful alternative procedure for portosystemic shunt procedures.

## Case presentation

The patient was 26-year-old man with medical history of recurrent upper gastrointestinal (GI) bleeding. There was no alcohol abuse in patient history. Endoscopic band ligation was performed for the first bleeding episode before this admission. On physical examination, findings related to chronic liver disease were not observed except splenomegaly. Pulse rate of 120 with a blood pressure of 110/80 mm Hg was found. The patient was also examined by endoscopically and inactive gastric varices and esophageal variceal re-bleeding were diagnosed. Liver function tests were normal and the other laboratory data were as follows; hematocrit level: 30%, erythrocyte count: 3400 × 10^3^/mm^3^, white blood cell count (WBC): 1500/mm^3 ^and platelet count: 31000/mm^3^. Prothrombin time (PT) and International normalization Ratio (INR) was mildly elevated (18.4 sec. and 1.49, respectively). The patient had a duplex ultrasound (DUS) examination of the abdomen that revealed the findings of PHT, splenomegaly, normal liver and no ascites. The diameter of portal vein was 20 mm with hepatopedal flow and left gastric vein (coronary vein) was 10 mm with hepatofugal flow. The patient also underwent Magnetic Resonance Portography (MRP) for further evaluation of portal venous anatomy. This revealed that; the diameter of splenic vein (SV) was 30 mm, left renal was 10 mm and there were retroperitoneal spontaneous shunts. Liver biopsy was also performed and pathological examination revealed nonspecific morphological changes  in small portal tracts, portal vein branches were inconspicuous. Extrahepatic non-cirrhotic PHT was diagnosed and DSRS was planned  to prevent esophageal variceal re-bleeding.

## Surgical technique

**Figure 1:**
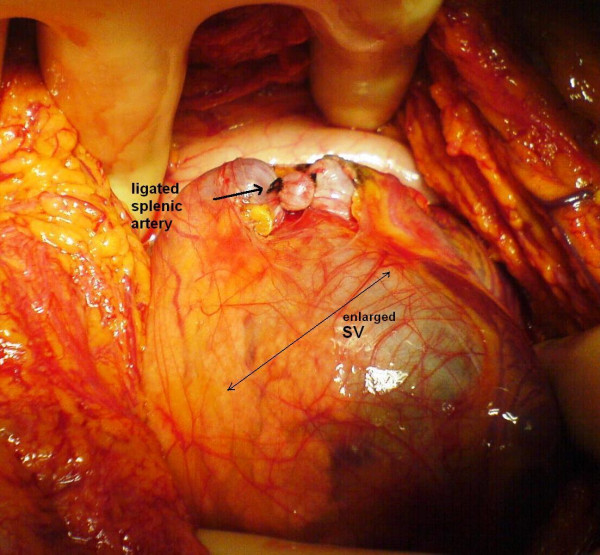
Enlarged splenic vein and ligation of splenic artery due to difficult dissection.

**Figure 2:**
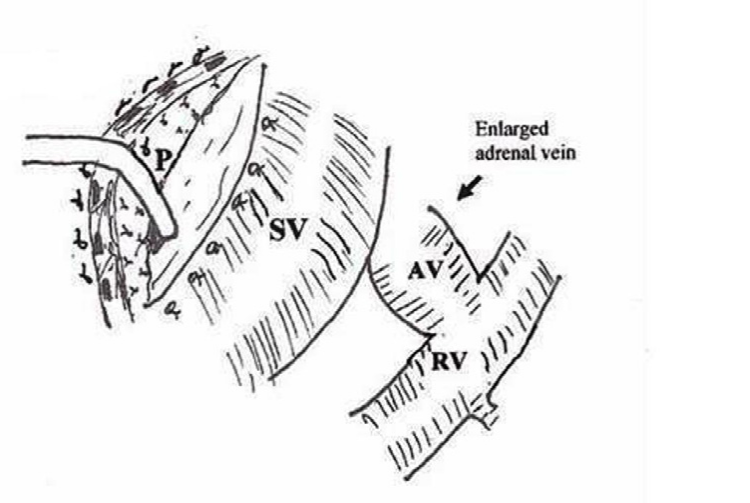
Splenic vein is isolated and separated from pancreas and surrounding tissue (P: Pancreas, SV: Splenic vein, AV: Adrenal vein, RV: Renal vein.).

**Figure 3:**
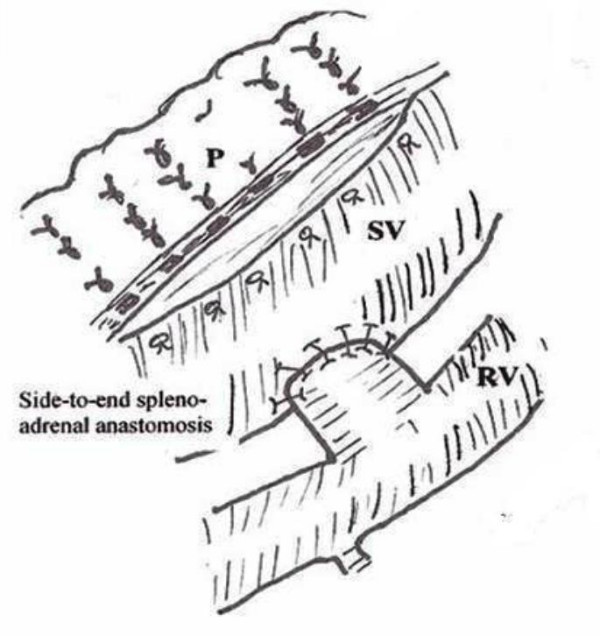
Anastomosis between SV and enlarged adrenal vein stumpy.

**Figure 4:**
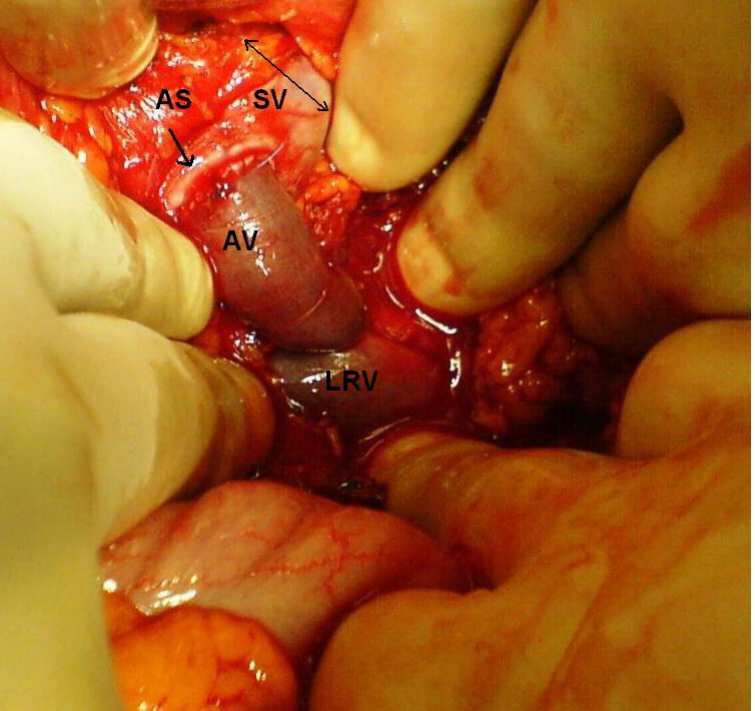
Completed portosystemic shunt using adrenal vein (AV) as a conduit between splenic vein (SV) and left renal vein (LRV). (AS = anostomosis site)

Laparotomy was performed through a left subcostal incision. No ascites was observed in the peritoneal cavity and the liver appeared normal. Nevertheless, the enlarged spleen was identified during the operation. Firstly, division of the gastrocolic ligament was performed and extended distally to obtain adequate view of the lower border of the pancreas. The splenic vein, left renal vein (LRV) and left adrenal vein were then visualized. Because it was quite difficult to achieve complete separation of SV from the pancreas due to surrounding dense, fibrotic tissue, end-to-side splenorenal anastomosis was not observed as an appropriate surgical technique. However, an excessive enlargement (4.5 cm diameter) and angulation of SV were noted. Since the portal pressure (PP) was measured 34 mm Hg, splenic artery was ligated in order to prevent likelihood massive bleeding during hard and risky dissection (figure 1). After then, PP was measured 26 mmHg. Adrenal vein stump measuring 1.2 cm in diameter was thought to be an alternative conduit between the SV and LRV (figure 2). Side-to-end spleno-adrenal vein anastomosis was performed in a running fashion with 6/0 polypropylene (figure 3, 4). Left gastric vein was also dissected and ligated. After shunt procedure was performed, PP was measured 16 mm Hg.

Postoperative course was unremarkable. Ammonia level after the shunt procedure was normal. The patient was discharged from hospital on the postoperative day 6 and followed up for nine months period by ambulatory. He had no complaint and had normal daily activities. DUS for shunt patency and upper GI endoscopic examinations for esophageal varices which may need to be treated were both normal.

## Discussion

DSRS as a selective porto-systemic shunt procedure is more effective and has less complication rate than both endoscopic therapy and TIPSS for the treatment and prevention of esophageal variceal bleeding due to both cirrhotic and non-cirrhotic PH [3,10]. It does not alter the liver function in extrahepatic portal hypertension (EHPH) [11]. EHPH is defined as extrahepatic hypertension of the portal venous system in the absence of liver cirrhosis. Although the endoscopic therapy, liver transplantation, and TIPSS have resulted in improved results for patients treated surgically for variceal bleeding, portosystemic shunts remain important and effective options for selected patients with bleeding secondary to portal hypertension. Whether TIPSS is as efficacious as surgical shunts is rather unproven. Its main drawback is still a high rate of restenosis or occlusion requiring frequent reinterventions [12].

Recurrent variceal bleeding which is one of the most important complications of PHT isn't uncommon even after medical and endoscopic management in EHPH [10]. Since repeated investigation of both endoscopic sclerotherapy and band ligation are often required, the patients recommended endoscopic treatment should be in a close contact with medical center. Nevertheless, the patients living in rural area as our case have a disharmony for this kind of therapy as cited in the literature [13]. Thus, surgical shunt therapy, particularly DSRS, is a popular palliative method to decompress PHT. It is an effective shunt procedure and does not usually need repeated intervention.

The main principle in shunt surgery is to create a tension-free and wide anostomosis with sufficient caliber, and thrombus-free veins to prevent obstruction of the shunt. Inappropriate anatomy (excessive fibrotic tissue, inadequate diameter of splenic vein e.g.) may cause decreased shunt patency that we suggest alternative shunt procedure avoiding hard maneuvers. Due to these anatomical challenges, prosthetic materials may be used or coronario-caval shunt or inferior mesenteric-left renal vein shunt can be employed in selected cases [3]. As we performed on this case, ligation of splenic artery leads to reduce diameter of the enlarged splenic vein. Thus, dissection of the vascular structures and managing of the end – to- side anostomosis can be performed much easier.

Adrenal vein as a conduit is another option to create a selective splenorenal shunt. DSRS via the adrenal vein has been used as an alternative technique in a few cases [5-9]. Because of the excessive fibrotic tissue surrounding SV and inadequate diameter between the SV and left RV to make a safe anastomosis, we preferred adrenal vein as a conduit to perform porto-systemic shunt. Because, Doppler examination of the shunt patency including flow pattern was optimal even after nine months later, any other diagnostic method for control was not needed. In case of suspected flow pattern with DUS, angiography may be useful to figure out the problem.

As in this case, in case of failure of DSRS because of technical problems, end-to side porto-systemic shunt procedure should be considered as an alternative method. For this procedure, prosthetic grafts, cadaveric vascular conduits, and autologous vascular graft as in this patient can be used. Since prosthetic grafts have a high risk for thrombosis causing occlusion and supplement the cadaveric vascular graft is difficult, adrenal vein as an autologous vascular graft should be kept in mind.

## Conclusion

In conclusion, in case of failure to perform routine surgical shunt procedure to decompress PHT, adrenal vein may be safely used as vascular conduit to create porto-systemic shunt procedure, particularly end-to-side splenorenal shunt in selected cases.

## Competing interests

There is no commercial association that might pose a conflict of interest in connection with this submitted communication which entitled "Porto-systemic shunt using adrenal vein as a conduit; An alternative procedure for spleno – renal shunt"

There are no sources of funding of the work.

## Authors' contributions

UA conceived of the study, and participated in its design and coordination, and managed the operation.

PY has made substantial contributions to acquisition of data and involved in drafting the manuscript.

MK participated in its design and gave the final approval of the version to be published.

All authors read and approved the final manuscript.

## Pre-publication history

The pre-publication history for this paper can be accessed here:


